# Synovial Chondromatosis of the Elbow: A Case Report and Discussion of Feared Complications

**DOI:** 10.7759/cureus.104415

**Published:** 2026-02-27

**Authors:** Sarthak Chakravarty, Kyle Colvin, Yassen Mubarak, Graham F Girdler, Gary Schwartz

**Affiliations:** 1 Orthopedic Surgery, Nova Southeastern University Dr. Kiran C. Patel College of Osteopathic Medicine, Fort Lauderdale, USA; 2 Orthopedic Surgery, Nova Southeastern University Dr. Kiran C. Patel College of Allopathic Medicine, Fort Lauderdale, USA; 3 Orthopaedic Surgery, Nova Southeastern University Dr. Kiran C. Patel College of Allopathic Medicine, Fort Lauderdale, USA

**Keywords:** arthroplasty, case report, open synovectomy, synovial chondromatosis, synovial chondrosarcoma

## Abstract

Synovial chondromatosis (SC) is a rare, benign neoplasm of synovium-lined joints that commonly presents with decreased mobility, pain, and swelling. It is believed to arise from mesenchymal cells that undergo cartilaginous metaplasia, the histological hallmark of the disease. There is a concern for malignant transformation to synovial chondrosarcoma (SCH). We report a case of SC with a subsequent literature review regarding malignant transformation. A 69-year-old male presented with a progressive history of right elbow pain and stiffness. On examination, the patient had significant limitation of range of motion (ROM) and tenderness of the elbow. X-rays and MRI showed findings suggestive of SC. This was treated operatively with removal of the loose bodies, and the patient was able to return to all his activities pain free. The Boolean search strategy of PubMed was utilized to include articles from 1980 to 2025. A total of 611 articles were identified, which were narrowed to 17 after blinded tier one and two screenings by three authors utilizing Rayyan. The extracted data included the mean patient age/number of patients, history, affected joints, procedures performed, SC size, follow-up, recurrence, timeline for malignant progression, and treatment used for SCH. Our review found that although plain radiograph is used most for initial assessment, computed tomography is the optimal modality to determine the characteristics of the intra-articular calcifications, while magnetic resonance was best suited for determining extension into surrounding tissues. Of the surgical interventions used, arthroscopy and synovectomy were most used in first-time occurrences. Those who develop recurrence or malignant transformation seek more aggressive intervention such as hemipelvectomy, laminectomy, or amputation. SC can normally be successfully treated with synovectomy. However, it is necessary to balance minimally invasive treatment and to reduce the risk of recurrence or malignant transformation.

## Introduction

Synovial chondromatosis (SC) is a rare, locally proliferative neoplasm that originates in the intra-articular space of a joint capsule arising from the synovial tissue or the bursa [1]. It is thought that the condition stems from the proliferation of mesenchymal cells, which subsequently undergo cartilaginous metaplasia. In long-standing cases, portions of primary growth can detach and become free bodies in the intra-articular space. Ossification can occur, causing pain and swelling. Joint mobility can also become impeded. The free bodies create a characteristic appearance in radiologic studies; however, the presence of cartilaginous metaplasia on histology is considered definitively diagnostic. The incidence is estimated at 1.8 in 100,000 people. It most commonly occurs in weight-bearing joints. Approximately 70% of cases occur in the knee or the hip; however, it can happen in any joint [2]. SC can be divided into primary and secondary forms. Primary SC's pathogenesis is unknown, but it is hypothesized that undifferentiated mesenchymal stem cells multiply in the synovium, forming localized foci of cartilage. Secondary SC occurs as a result of another joint pathology, such as osteoarthritis, trauma, inflammatory arthropathies, or avascular necrosis, which can cause cartilage fragments to embed into the synovium [2]. Management is usually a synovectomy, either open or using arthroscopy, depending on presentation. It is noted that in the hip cases where the peripheral and central zones are both involved, open arthrotomies may lead to lower levels of recurrence. The difficulty of full access to the intra-articular space is believed to contribute [3]. In the knee, total knee arthroplasty is effective; however, it is also associated with the potential for recurrence [4].

A feared complication of SC is the potential for malignant transformation into synovial chondrosarcoma (SCh). In patients with SC, the rate of progression to SCh is estimated at 5 percent or less [5]. Progression to SCh is associated with high-grade SC in cases of recurrence. In patients with long-standing SC, accelerated progression of symptoms should alert clinicians to the possibility of malignancy [4]. Patients with SCh are treated aggressively, with bone resection and potential amputation of the affected limb. Chemotherapy and radiation can be used, but are associated with lower survival rates.

In this article, we present a case report of SC of the elbow and perform a review of the literature with regard to progression to SCh.

## Case presentation

**Figure 1:**
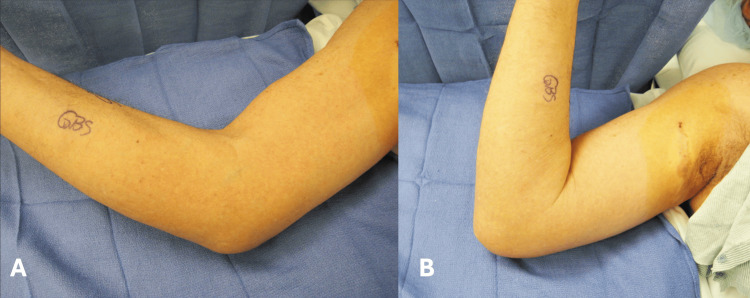
Pre-operative active and passive range of elbow motion Pre-operative active range of elbow extension (A) and (B) active range of elbow flexion

A 69-year-old man presents with a chief complaint of pain and limited motion in the right elbow for three years. There was no history of trauma or previous similar episodes. The symptoms have been progressive in nature and interfering in his daily activities. He was an avid golfer and encountered multiple episodes of locking of his elbow, as well as stiffness after periods of inactivity. He noted a baseline level of stiffness present in his right elbow that was not present on his left. These symptoms were interfering with his ability to play golf pain-free. On examination of the right elbow, active and passive range of motion was from 70 to 110 degrees (Figure 1). There was mild swelling present about the elbow joint, and there was global tenderness primarily with terminal elbow flexion and extension. Upon deep palpation of the antecubital fossa, a nodular mass was noted. 

**Figure 2:**
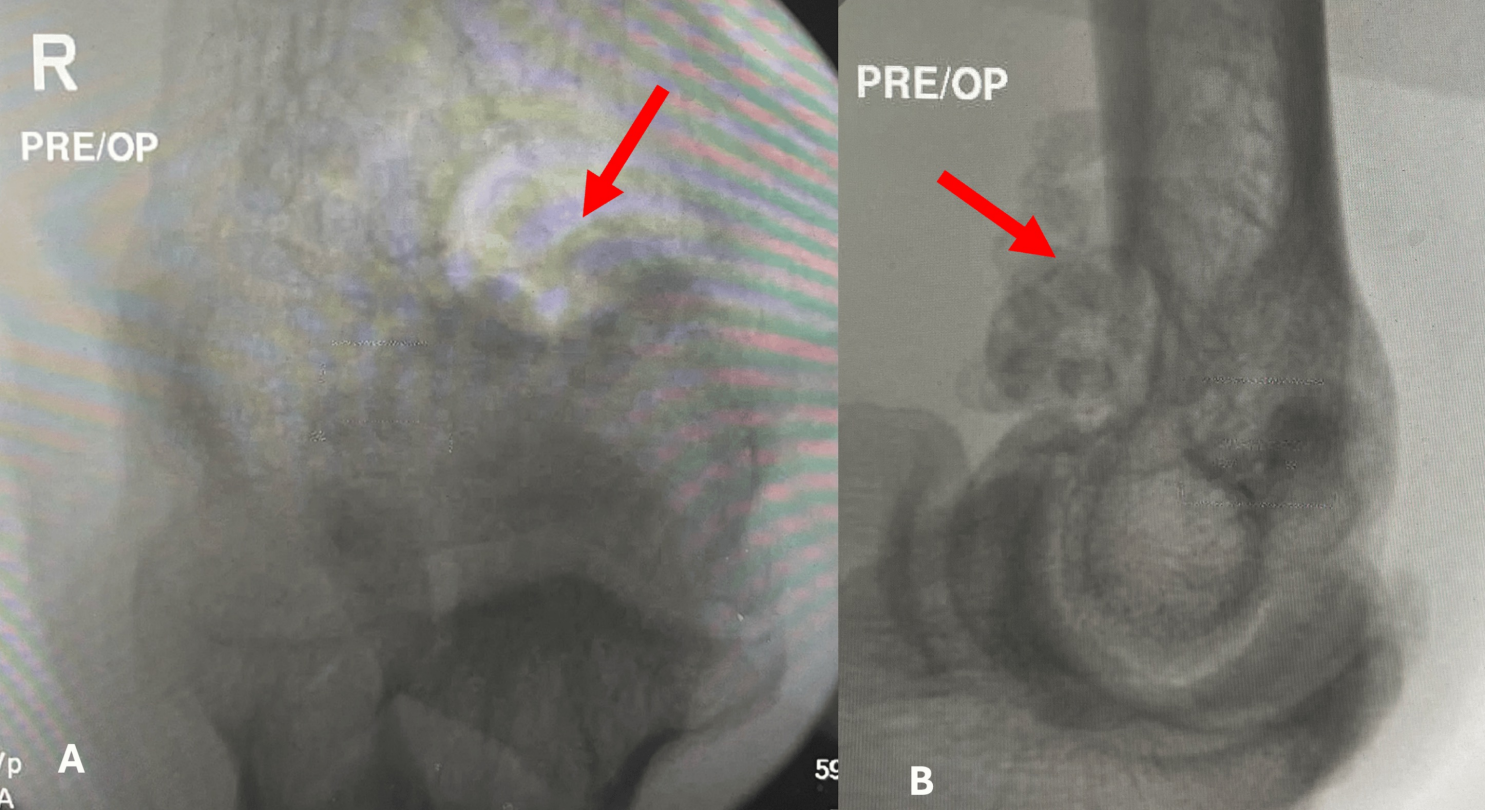
Pre-operative radiographs of the right elbow Pre-operative (A) anterior-posterior and (B) lateral radiographs of the right elbow demonstrating multiple loose bodies in the elbow joint (red arrows).

Radiographs of the right elbow demonstrated multiple opaque loose bodies in the elbow joint (Figure 2).

**Figure 3:**
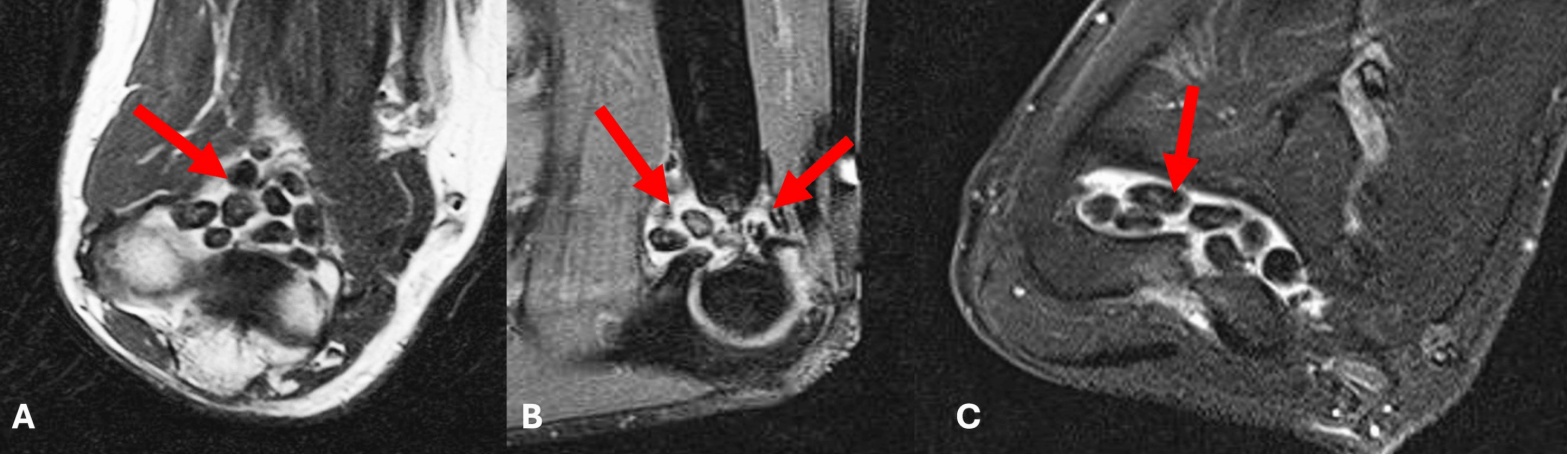
Pre-operative magnetic resonance imaging (MRI) of the right elbow MRI in the (A) coronal, (B) sagittal, and (C) axial planes demonstrate multiple loose bodies in the elbow joint (red arrows).

An MRI of the right elbow was ordered due to the significant swelling and stiffness, and to further characterize the mass. This demonstrated innumerable well-circumscribed ossific fragments within the elbow joint measuring up to 14 mm in dimension. There was mild elbow joint osteoarthritis associated with joint space narrowing and marginal osteophyte formation, most notably involving the ulnotrochlear joint. Most of the loose bodies were in the anterior aspect of the distended joint capsule. There were several within the olecranon fossa (Figure 3) (red arrows). The patient's clinical history, in conjunction with these findings on advanced imaging, prompted the diagnosis of SC.

**Figure 4:**
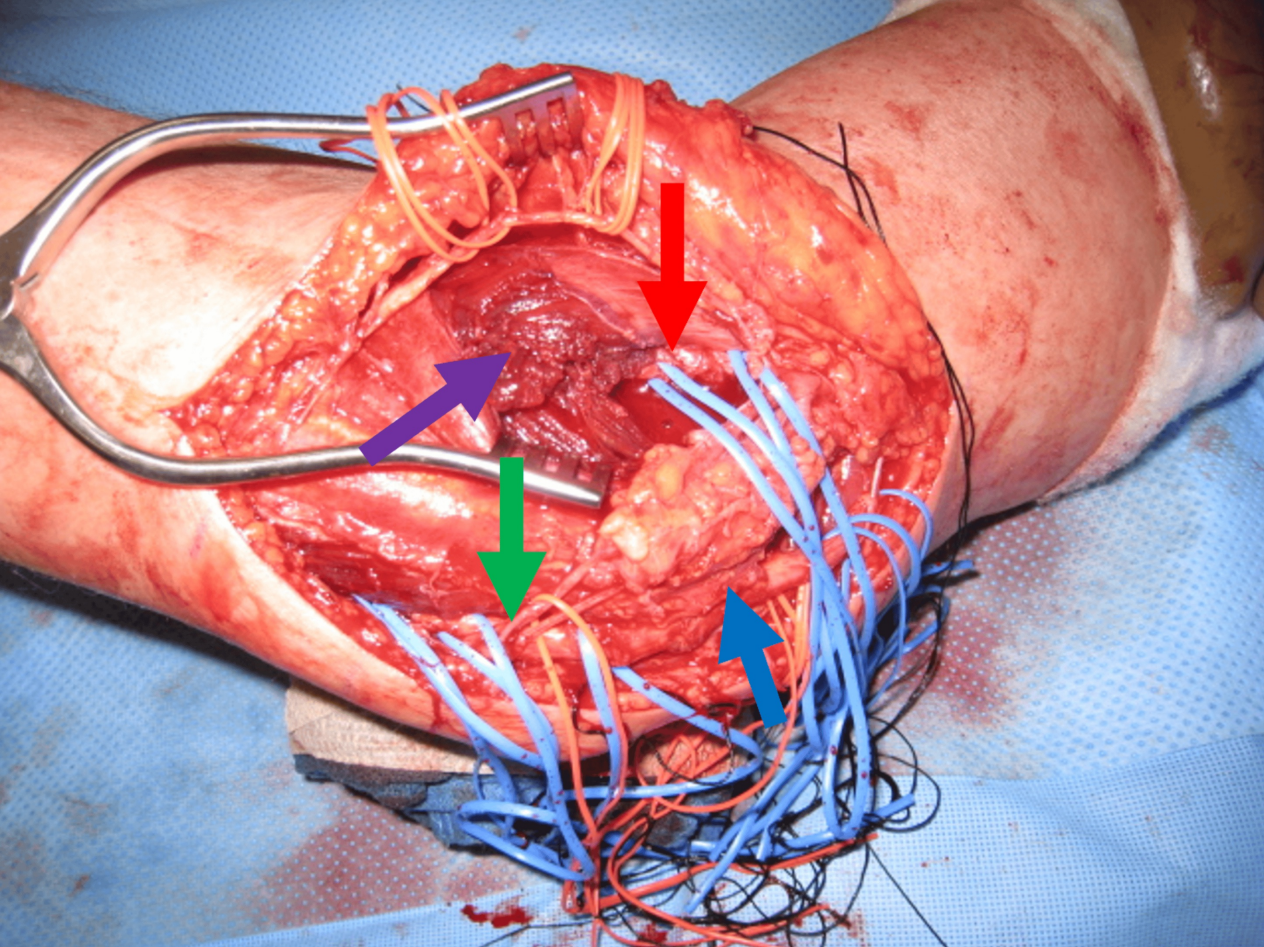
Intra-operative photo of the right elbow Intra-operative photo of the right elbow demonstrating the medial approach, identifying the ulnar nerve (blue arrow), medial antebrachial cutaneous nerve (green arrow), the median nerve (red arrow), and the reflected flexor pronator mass (purple arrow).

Due to the interference in the patients' daily activities, an open synovectomy was indicated for the release of the elbow contracture with the removal of the multiple loose bodies. The elbow was approached through a medial incision. This extended from 8 cm proximal to 8 cm distal to the medial epicondyle. The medial antebrachial cutaneous and ulnar nerves were identified and protected. A portion of the medial intermuscular septum was excised. An incision in the midline of the flexor pronator mass was made to identify the median nerve. The brachialis was swept off the anterior capsule, which was incised from a lateral to a medial direction (Figure 4).

**Figure 5:**
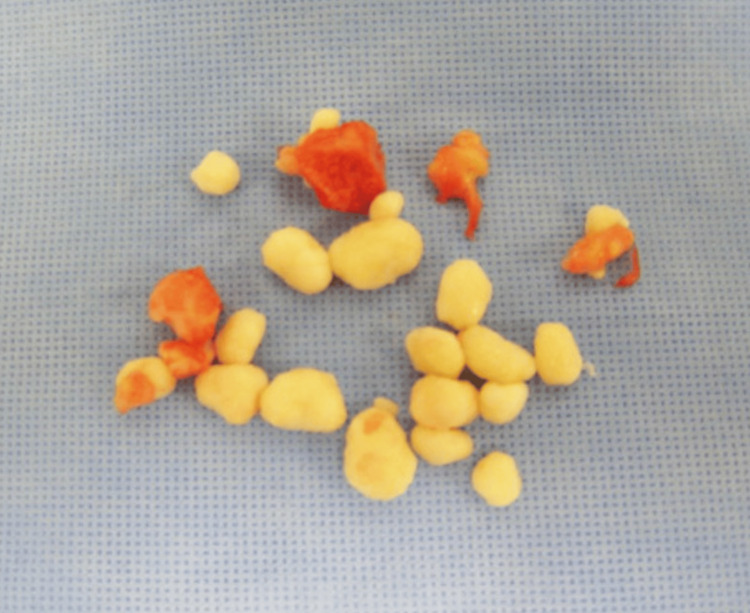
Specimen removed from the right elbow Multiple loose bodies removed from the right elbow.

The ulnar nerve was transposed anteriorly, and the interval between the humerus and olecranon was identified. Multiple loose bodies were identified both in the anterior and posterior aspects of the elbow joint. These were removed (Figure 5).

**Figure 6:**
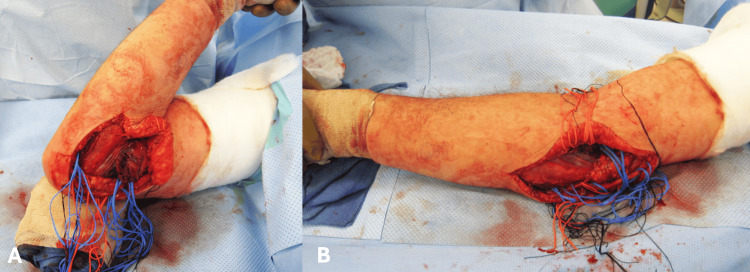
Elbow motion at the end of the procedure The motion at the termination of the procedure was from 10 to 130 degrees.

At the end of the operative procedure, the passive range of motion was from 10 to 130 degrees of flexion (Figure 6).

The patient was referred to occupational hand therapy for a period of four months. At that time, the patient was discharged from active orthopedic care. The patient returned to all of his activities without any further symptoms. The active elbow motion during follow-up to the time of discharge was maintained at 10-130 degrees of active flexion.

## Discussion

Methods

Preliminary articles were selected during an electronic search of one peer-reviewed literature database (PubMed) published from 1980 to 2025. The Boolean search strategy used combined search terms as follows: “synovial chondromatosis” AND "recurrence”, “synovial chondromatosis” AND “total synovectomy”, “synovial chondromatosis” AND “partial synovectomy”, “synovial chondromatosis” AND “arthrodesis”, “synovial chondromatosis” AND “radiation therapy” OR “radiation treatment” AND “recurrence”, “synovial chondromatosis” AND “synovial chondrosarcoma”, “synovial chondromatosis” AND “surgery” AND “recurrence”. Articles screened from the literature search were managed using Rayyan (Rayyan Systems Inc., California, MA), an online software designed for screening large volumes of literature for reviews. 

The inclusion criteria for our studies included case reports, randomized controlled trials, retrospective studies, cohort studies, or longitudinal studies in the English language that directly discussed SC, as well as those that discussed recurrence of SC or progression to synovial chondrosarcoma. Articles addressing other bone tumors without the presence or history of SC, involving non-human patients, involving the temporomandibular joint, or lacking the full text were excluded, alongside duplicates and any article that did not otherwise meet the inclusion criteria.

**Figure 7:**
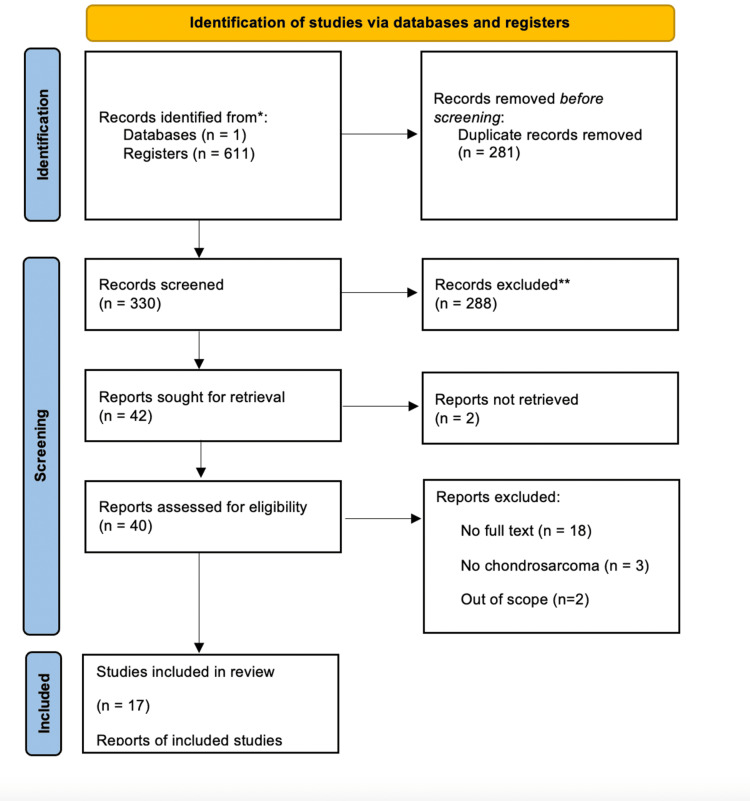
Preferred Reporting Items for Systematic Reviews and Meta-Analyses (PRISMA) flow diagram detailing the process of screening studies. The figure details the process of sorting studies based on the PRISMA guidelines [6]. This includes a deduplication process, along with Tier 1 and Tier 2 screening.

The screening was completed in accordance with PRISMA guidelines [6]. After the initial literature search, the total number of articles in our screening pool was 611. After deduplication and removal of papers that fit, with the above exclusion criteria, there were a total of 330 articles. A blinded Tier 1 screen was completed by three of the authors via reading through titles and abstracts and eliminating articles that did not fit the inclusion criteria. After Tier 1 screening, there were a total of 42 articles that continued to Tier 2 screening. Three authors completed the Tier 2 screening by reading the full texts of the articles and sorting by inclusion criteria. After full-text screening, there were 17 articles that were included in the final study (Figure 7).

**Figure 8:**
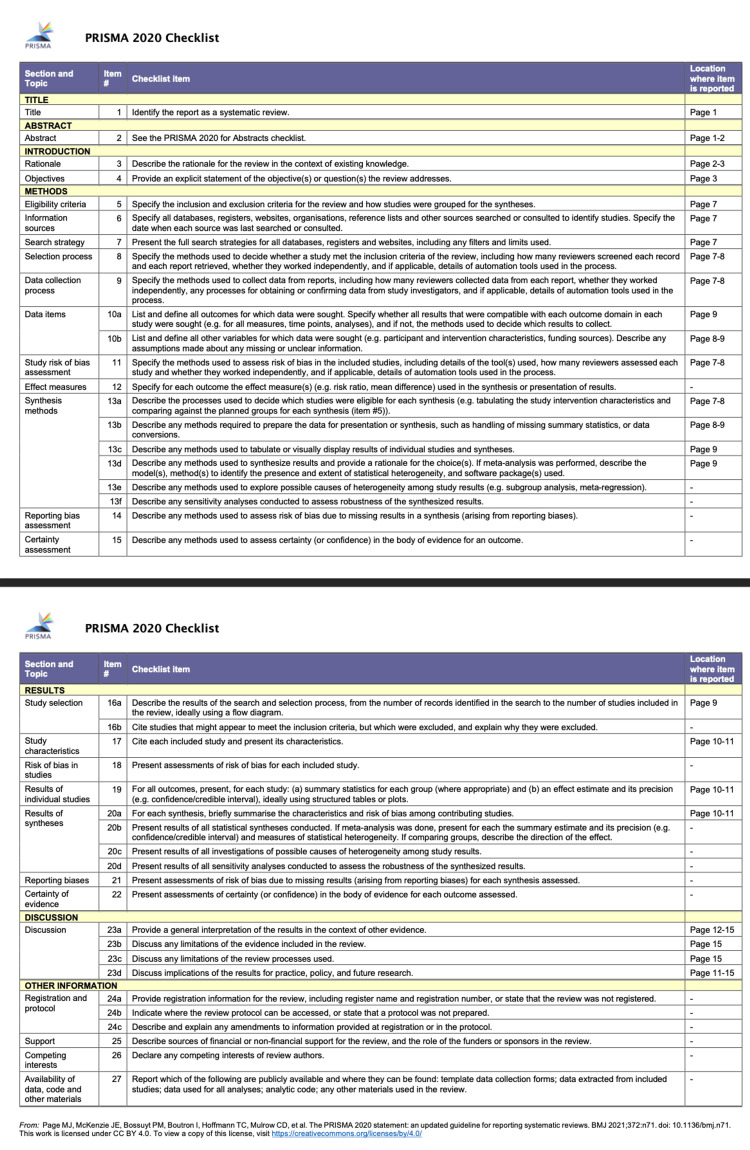
A Preferred Reporting Items for Systematic Reviews and Meta-Analyses (PRISMA) 2020 checklist, with page numbers listed corresponding to the criteria met. If not applicable, this is denoted with a "-".

Data were extracted manually from the 17 included articles by searching for mean patient age/number of patients, history/symptoms, affected joints, procedures done, SC size, follow-up, recurrence, timeline for malignant progression, and treatment used for SCH (chondrosarcoma). Some findings within the body of the literature review were also noted in Bertoni et al's study, which was one of the first articles that studied SC and transformation to SCH. A table of the articles selected, listed by author, study title, and joint studied, is listed below (Table 1) [7-24]. A PRISMA checklist was completed to ensure rigorous reporting of the data acquired (Figure 8).

**Table 1:** Final 17 studies included in our literature review. Listed by authors, title of their study, and the joint studied.

Authors	Title	Joint Studied
Galat, Daniel D et al. [7]	Synovial Chondromatosis of the Foot and Ankle	Ankle (n = 6), midfoot (n = 2)
Furtado, Cleofina et al.^ [8]^	Secondary Chondrosarcoma Arising in Synovial Chondromatosis of Wrist Joint	Wrist
Wenger, D E et al. [9]	Acral Synovial Chondrosarcoma	Distal first metacarpal and base of the proximal phalanx (n = 1)
Littrell, Laurel A et al. [10]	Chondrosarcoma Arising Within Synovial Chondromatosis of the Lumbar Spine	Right L4-5 facet joint
Urwin, John W et al.[11]	Malignant Transformation of Recurrent Synovial Chondromatosis: A Case Report and Review	Left hip
Taconis, W K et al.[12]	Synovial Chondrosarcoma: Report of A Case and Review of the Literature	Right hip
Rybak, Leon David et al. [13]	Primary Synovial Chondrosarcoma of the Hip Joint in a 45-Year-Old Male: Case Report and Literature Review	Left hip
Campanacci, Domenico Andrea et al. [14]	Synovial Chondrosarcoma of the Hip: Report of Two Cases and Literature Review	Hip (n = 2)
McCarthy, C et al.[15]	Primary Synovial Chondromatosis: A Reassessment of Malignant Potential in 155 Cases	Hip and elbow
Hermann, G et al.[16]	Synovial Chondrosarcoma Arising in Synovial Chondromatosis of the Right Hip	C7
Kyriazoglou, Anastasios I et al. [17]	Cytogenetic Analysis of a Low-Grade Secondary Peripheral Chondrosarcoma Arising in Synovial Chondromatosis	Left wrist
Bhadra, A K et al.[18]	Primary Tumours of the Synovium. A Report of Four Cases of Malignant Tumour	Right knee
Murphey, Mark D et al. [19]	Imaging of Synovial Chondromatosis With Radiologic-Pathologic Correlation	Knee is the most common
Wang, Wei et al.[20]	Clinical Comparison of Tenosynovial Giant Cell Tumors, Synovial Chondromatosis, and Synovial Sarcoma: Analysis and Report of 53 Cases	LE (n = 12), hip (n = 2), UE (n = 2)
Amary et al.[22]	Synovial Chondromatosis and Soft Tissue Chondroma: Extraosseous Cartilaginous Tumor Defined by FN1 Gene Rearrangement	Knee (n = 47), then hand/wrist, hip, elbow, foot/ankle, and shoulder
Agaram, Narasimhan P et al.[23]	A Molecular Study of Synovial Chondromatosis	Knee (n = 14), finger (n = 3), wrist (n = 3)
Gambarotti, Marco et al.[24]	Synovial Chondrosarcoma: A Single-Institution Experience With Molecular Investigations and Review of the Literature	Knee (n = 5), hip (n = 2), glenohumeral joint (n = 1), foot (n = 1), ankle (n = 1)

Another summary table detailing some key findings, such as duration of patient symptoms, imaging findings, treatment used, and follow-up (if available), is detailed below (Table 2). 

**Table 2:** Some key findings of patients from the included studies. These include duration of symptoms, imaging findings (XR, CT, US, and MRI), treatment options pursued, and status after follow-up.

Authors	Duration of symptoms	Imaging findings	Treatment used	Follow-up (if available)
Galat, Daniel D et al. [7]	Four years after initial excision	Not described	Ankle synovectomy with loose body removal (n = 4), excision and midfoot arthrodesis (n = 1), below-knee amputation (n = 3).	Mean of 9.5 years
Furtado, Cleofina et al. [8]	Seven year hx of symptoms before seeking evaluation	Case 1: enlarging periarticular soft-tissue swelling with internal mineralization at the right wrist with larger bone erosions as compared to previous X-rays. A subsequent MRI demonstrated profuse synovial proliferation into large peri-articular soft tissue or synovial masses. Case 2: Radiographs were negative. US showed a solid lobular lesion on the volar-radial aspect of the wrist, displacing the flexor tendons and showing no evidence of internal vascularity. MRI showed invasion of the soft tissue mass into neighboring tendons.	Case 1: Initial plan to debulk disease, ultimately led to below-elbow amputation because of malignancy. Case 2: excisional biopsy.	N/A
Wenger, D E et al. [9]	Three months of symptoms	Radiographs showed scalloped destructive lesions on the medial and volar aspects of the distal first metacarpal and base of the proximal phalanx with the two bones showing relatively equal destruction. The joint margin and joint space were preserved. MRI showed a soft tissue cartilaginous mass.	Wide resection with thumb allograft placement	18 months, disease-free
Littrell, Laurel A et al. [10]	10 years of lower back pain	XR showed a mineralized soft tissue mass along the posterior lower lumbar spine. CT better characterized the calcifications. MRI showed large, heterogeneous soft- tissue mass with irregular margins that insinuated into the neural foramina.	Staged wide resection of the tumor with partial corpectomies at L4 and L5, right hemilaminectomies from L3-S1, and posterior instrumented fusion from L2 through sacrum and ilium	Five years disease-free
Urwin, John W et al. [11]	Patient experienced a 1 year history of left hip pain and mass.	XR showed soft tissue calcifications with erosion. CT better characterized the calficiations and articular involvement.	Open subtotal debulking, followed by THA one year later due to malignancy, then repeat debulking two months subsequently.	N/A
Taconis, W K et al. [12]	Patient had a 6 year hx of symptoms, worsened in the last 2 years.	XR showed faint soft tissue swelling with multiple round to ovoid calcifications. CT showed the erosion and faint calcifications. MR better classified the invasiveness of the tumor.	Open synovectomy	Lost to follow-up for four years. He was reevaluated for paresthesias of the right upper extremity (RUE) and right lower extremity (RLE). The patient passed away three months later.
Rybak, Leon David et al. [13]	5 year history of symptoms	XR showed irregularly shaped peri-articular ossific densities with erosion. MRI showed lobular soft tissue in the joint space with excavation of surrounding bony components.	THA initially, followed by acetabular excision and reconstruction, and placement of a long-stem femoral implant	N/A
Campanacci, Domenico Andrea et al. [14]	Case 1: intermittent hip pain, with a 3 week history of worsening Case 2: 10 year history of hip pain	XR showed irregular calcific masses with bony erosions. CT and MRI showed thickened synovium containing these calcified bodies with further bony erosion.	Case 1: Incisional biopsy followed by external hemipelvectomy. Case 2: Palliative radiation	Case 1: Disease-free 10 years post-op. Case 2: alive with disease 58 months after diagnosis.
McCarthy, C et al. [15]	Case 1: Recurrence within 6 months of initial procedure (THA+synovectomy) Case 2: 3 year history of stiff and painful elbow Case 3: not available Case 4: not available	Peri/intra-articular calcific mass (XR), multiple foci of low signal calcification, cortical destruction of the acetabulum with intramedullary bone infiltration (MRI), mild–moderate FDG uptake with a maximum standardised uptake value (SUV) of 4.1 (FDG-PET).	Case 1: THA +synovectomy with three debulking procedures afterward. Case 2: Synovectomy with repeat excision + arthrolysis after three months. Case 3: Hemipelvectomy +endoprosthetic reconstruction. Case 4: Anterior resection of the mass.	Case 1: not available. Case 2: no recurrence in two years post-op. Case 3: no recurrence in two years post-op. Case 4: no recurrence in three years post-op.
Hermann, G et al. [16]	2 month history of neck pain 2 years after having surgery for SC of the hip.	Radiographs unremarkable. MRI showed destructive process of C7 with suspicion for cord compression.	Decompressive laminectomy, followed by vertebral resection	The patient experienced a pulmonary embolism and passed away.
Kyriazoglou, Anastasios I et al. [17]	10 year history of growing movable mass of the wrist.	Computer tomography (CT) scan revealed a lesion in the left dorsal region of the cubitus.	Total resection of the tumor, followed by external fixation	Not available
Bhadra, A K et al. [18]	6 month history of knee swelling and stiffness	Radiographs and subsequent MRI showed "popcorn" calcification consistent with synovial chondromatosis.	Open debulking, followed by above-knee amputation three months afterward	Alive six months post-op

Results

Imaging Findings

We began analyzing the articles based on imaging findings, the surgical interventions chosen, and molecular studies. There were similar themes that arose among imaging findings across the different modalities used [7-18]. In general, plain radiographs were the initial diagnostic modality used to investigate their patients' symptoms. The findings on X-ray imaging among these studies were quite similar for SC across multiple joints. These included periarticular soft tissue swelling with foci of calcification/mineralization. There were also findings of joint erosion/destruction with preservation of the joint space noted. Computed tomography was useful in determining the extent of joint erosion, as well as the nature of the calcifications (intra/extra/periarticular). MRI of SC showed some characteristic findings across the studies, namely, low signal intensity on T1-weighted images and high signal intensity on T2-weighted images. Fat-saturated sequences showed SC as a bright white mass. When signal dropout was present on any sequence, it was suggestive of calcifications [7]. Multiple studies showed evidence of invasion to neighboring structures on MRI [8-10,13,15,16]. Findings included mass effect and splaying of flexor tendons of the wrist [8], joint effusion [9], entering neural foramina in the lumbar spine [10], excavation of multiple areas of the hip joint [13], infiltration into surrounding musculature [15], and findings suspicious for spinal cord compression in the cervical spine [16]. Gadolinium-enhanced MR showed areas of solid heterogeneous enhancement and scattered areas of non-enhancing myxoid change with punctate nodular peripheral enhancement, compatible with a cartilage tumor [10]. 

Campanacci et al. were the only ones of our reviewed studies that performed a radionuclide bone scan, which showed increased focal uptake in the area of concern [14]. Similarly, McCarthy et al. were the only ones of our reviewed studies who completed an FDG-PET scan, which showed a mild to moderate FDG uptake with a maximum standardized uptake value of 4.1 [15]. 

Murphey et al.’s [19] study describes the pros, cons, and diagnostic pearls associated with the different types of imaging studies when a case of SC is suspected. Typically, radiographs reveal multiple intra-articular calcifications in 70-95% of cases of primary SC, and the calcifications are typically distributed evenly throughout the joint. They also usually display a chondroid ring-and-arc pattern of mineralization, and mineralization is more likely to develop with longer-standing disease. Chronic disease may also lead to secondary osteoarthritis and joint space narrowing in the joints, such as the shoulder and hip, although typically the joint space is maintained. Secondary SC tends to involve fewer intra-articular fragments that are more variable in size, as well as an underlying pathology present on radiographs (most commonly osteoarthritis) [19].

Ultrasound tends to show a heterogeneous mass containing foci of hyperechogenicity, which can represent the chondral fragments within the joint, bursa, or tendon sheath. CT scanning is the optimal imaging modality to detect and characterize calcification, and the vast majority of cases of primary SC reveal this feature. Erosion of the bone is also optimally imaged with CT. MRI will most frequently reveal a lobulated, homogeneous, intermediate, intra-articular signal intensity similar to that of muscle on T1-weighted images, with high signal intensity on T2-weighted images [19].

Regarding determining progression to synovial chondrosarcoma, extension into the adjacent soft tissues (particularly into bursae) may be seen both with and without malignant transformation and is therefore not feasible to distinguish between the two conditions. The same can be said for extrinsic erosion of the bone. True cortical destruction with bone marrow invasion and permeation are features that are suspicious for malignancy, and MRI is the modality that can best be used to detect such changes [19]. 

Surgical Interventions Chosen

One of the major decisions in the case of SC involves the type and extent of treatment used. Treatments used range from a relatively simpler excision biopsy to arthroscopic and open techniques, and then more invasive and considerably more serious treatments such as hemipelvectomy and amputation. Arthroscopy or simpler procedures, such as synovectomies, were typically used for first-time occurrences of SC. Arthroplasty appeared to be the middle ground between conservative and aggressive treatment. Of the four studies that treated patients with arthroplasty [11,13,15,20], two of them performed arthroplasty as their first surgical treatment [13,15], and two of them performed arthroplasty after a less invasive approach had failed [11,20]. The more aggressive or limb-sacrificing techniques (i.e., hemipelvectomy or amputation) were usually reserved for patients with multiple recurrences and cases of recurrent SC that had transformed to SCH [7,8,10,13-16,18]. One patient in the reviewed studies declined hemipelvectomy and opted to undergo palliative radiation and was alive with disease at 58 months post diagnosis before being lost to follow-up [14]. We reviewed two studies that discussed SC and chondrosarcoma of the spine [10,16]. Both studies treated their patients with decompressive laminectomies and resection of the vertebrae involved (L4-L5 and C7, respectively), with instrumented fusion performed in the patient with lumbar spine pathology. The patient in Hermann et al’s study presented with lower neck/upper back pain that, after investigation, was shown to be a metastatic lesion from a primary lesion of the hip. This patient was previously treated for SC of the hip 2 years prior with a THA. Review of the histology tissue from two years prior, with tissue taken from the C7 lesion, showed that both tissue samples showed synovial chondrosarcoma arising on a background of SC [16]. CT of the chest and pelvis revealed a mass in the posterior acetabulum and multiple nodular densities in the lungs, suggestive of metastasis. Typically, lung metastases are common (up to 56%) in patients with synovial chondrosarcoma [19,21]. In addition, metastasis to a distant bony site such as the cervical spine is also an unusual finding. 

Molecular Data/Histology

Differentiating between SC and synovial chondrosarcoma on tissue biopsy is key, as making one diagnosis over the other can have significant implications on the treatment choices made. First, on gross pathology, SC shows innumerable osteochondral bodies of similar size and shape with lobulated growth seen on hematoxylin and eosin (H&E) stain, similar to other hyaline cartilage neoplastic processes, but under the synovial lining [19]. On higher power zoom (200x), there are regions of synovial hyperplasia, with chondral bodies that appear blue on H&E, which are surrounded by pink enchondral bone formation. This pattern of multiple cartilage nodules surrounded by bone is responsible for the characteristic “ring and arc” appearance that is seen on X-Ray images of SC [19]. On 400x zoom, primary SC often appear hypercellular with atypical histologic features such as multinucleation, nuclear crowding and enlargement, hyperchromasia, and mild myxoid change [19,21]. 

While at first glance it may seem difficult to differentiate between SC and SCH, Galat et al. described some key differences that are present in synovial chondrosarcoma that are helpful in differentiating it from SC. First, in SCH, chondrocytes are arranged in a sheet-like appearance as compared to the clusters in SC. Second, there is a marked myxoid change in the matrix in chondrosarcoma compared to a solid matrix with SC. Furthermore, crowding and spindling of the chondrocytes is a sign of malignancy, with necrosis, bone invasion, and marrow permeation pointing one towards the diagnosis of Synovial chondrosarcoma over chondromatosis instead [7]. 

We reviewed five articles that studied SC and synovial chondrosarcoma at a molecular level [17,22,23,24,25]. Baumhoer et al noted that they identified FN1-ACVR2A and ACVR2A-FN1 fusions in two of their cases of chondrosarcoma arising on the background of SC. They report that 31 of 57 cases (54%) of SC and 2 of 3 cases of synovial chondrosarcoma harbor FN1 and/or ACVR2A gene rearrangements, as seen by FISH probe. They note that these alterations define these tumor types, but cannot differentiate between the two. They also note that mutations in isocitrate dehydrogenase (IDH) have been found in certain periosteal cartilaginous tumors, but never in SC, which can be a useful tool for ruling out SC as a possibility when a tumor exhibits an IDH mutation [25]. 

Agaram et al similarly noted FN1 and/or ACVR2A gene rearrangements in 67% of their cases. They also noted two novel mutations, FN1-NFATC2 and KMT2A-BCOR. The KMT2A-BCOR fusion occurred in a case of dedifferentiated synovial chondrosarcoma. The tumor that was sequenced was a 14x9x6.5 cm mass resected from the axilla that was abutting the proximal humerus, which was histologically described as dedifferentiated chondrosarcoma with areas of osteosarcoma arising in a background of SC (synovial dedifferentiated chondrosarcoma). Further analysis revealed an in-frame fusion between KMT2A exon 3 and BCOR exon 7 [23]. FN1 (Fibronectin 1) is a gene that encodes fibronectin protein, which is a soluble protein associated with cell adhesion and migration processes including embryogenesis, wound healing, blood coagulation, host defense, and metastasis [26], while ACVR2A encodes a transmembrane serine-threonine kinase receptor that mediates the functions of activins, which are members of the transforming growth factor-beta (TGF-beta) superfamily and have functions in skeletal development [23,27]. Regarding the KMT2A-BCOR fusion in the case of synovial chondrosarcoma, the KMT2A gene is a transcriptional coactivator that plays an essential role in regulating gene expression during early development and hematopoiesis, including many HOX genes [28], while the BCOR gene encodes a protein that acts as an interacting corepressor of BCL6, which may influence apoptosis [29]. 

Amary et al similarly found a high incidence of FN1-ACVR2A fusions in SC (57%) and in SCH (75%) as studied by FISH probe [22]. Additionally, their study described that RNA sequencing was able to find these alterations in several cases, which were negative by FISH. Their study also described variations in CDKN2A copy number in both SC and synovial chondrosarcoma. CDKN2A is a gene that leads to the synthesis of multiple tumor suppressor proteins. When they studied the samples of synovial chondrosarcoma, they found two showed homozygous deletion, one showed a copy number gain, and one (case 3) showed a disomic pattern in the SC component, but the chondrosarcoma component was unavailable for analysis. Eighteen cases of SC revealed disomy of CDKN2A [22].

Kyriazoglou et al found a t(1;14) translocation on their case of SC transforming into SCH, and they speculate that homeobox genes PBX1 or PRRX1 may have a role to play in progression of SCH, as the specific loci on chromosomes 1 and 14 have previously been shown to contribute to the development of chondrosarcoma and other solid tumor types [17]. 

## Conclusions

SC is a rare, locally proliferative neoplasm that originates in the intra-articular space of a joint capsule, arising from the synovial tissue or the bursa. It is thought that the condition stems from the proliferation of mesenchymal cells, which subsequently undergo cartilaginous metaplasia. We discussed a case report of a 69-year-old male with SC of the elbow, who was treated with open synovectomy and removal of the loose bodies. A feared postoperative complication of SC is recurrence, and more ominously, a transformation of the tissue to chondrosarcoma. Our literature review attempted to find trends in the management of patients with concern for SCH, as well as discussed the more common imaging and histological findings in the disease. We found that plain radiographs of the affected joint were typically the initial diagnostic tool used and showed an irregular soft tissue mass with calcifications present. We found that CT was the best modality to determine such calcification of the mass, while MRI was the best tool to determine the invasiveness and malignant characteristics of the tumor, such as invasion into surrounding structures. There was a wide range of surgical treatments used to treat SC and SCH. This ranged in invasiveness from simple excision biopsies to arthroscopic/open synovectomy, arthroplasty, and finally hemipelvectomy/amputation, which was reserved for multiple recurrences or SCH. One outlier from our reviewed studies was a patient who opted for palliative radiation therapy, who was alive with disease 58 months post diagnosis. This opens the discussion for the potential use of radiation therapy for the treatment of SCH. In addition, the presence of certain genetic and immunological patterns, such as FN1/ACVR2A gene rearrangements or the KMT2A-BCOR fusion, may suggest a role for medical treatment and genetic technologies as possible future treatment options for SC and SCh. Our study aimed to highlight the gaps in the literature with regard to considering SC and SCH as a diagnosis in patients with pain, stiffness, and/or a palpable mass in a joint. Care of these patients requires careful analysis of the patient's symptoms and timeline, thorough imaging review, as well as a multidisciplinary approach with close follow-up to monitor for signs of recurrence or malignant transformation. In order to better determine optimal treatment options for patients with recurrent SC and SCH, further research needs to be completed. 
